# The Marine Metabolite SZ-685C Induces Apoptosis in Primary Human Nonfunctioning Pituitary Adenoma Cells by Inhibition of the Akt Pathway *in Vitro*

**DOI:** 10.3390/md13031569

**Published:** 2015-03-23

**Authors:** Xin Wang, Ting Tan, Zhi-Gang Mao, Ni Lei, Zong-Ming Wang, Bin Hu, Zhi-Yong Chen, Zhi-Gang She, Yong-Hong Zhu, Hai-Jun Wang

**Affiliations:** 1 Department of Histology and Embryology, Medical school of Sun Yat-sen University, No.74, Zhongshan Road 2, Guangzhou 510080, China; E-Mails: wangx357@mail2.sysu.edu.cn (X.W.); tanting395395@sina.com (T.T.); leini@mail2.sysu.edu.cn (N.L.); cesshzhg@mail.sysu.edu.cn (Z.-G.S.); 2 Department of Neurosurgery and Pituitary Tumour Center, The First Affiliated Hospital of Sun Yat-sen University, No.74, Zhongshan Road 2, Guangzhou 510080, China; E-Mails: mmh222111@aliyun.com (Z.-G.M.); wzmyan@gmail.com (Z.-M.W.); 13512725071@163.com (B.H.); chenzhiyong111@126.com (Z.-Y.C.); 3 Key Laboratory of Functional Molecules from Marine Microorganisms, Department of Education of Guangdong Province, Sun Yat-sen University, No.74, Zhongshan Road 2, Guangzhou 510080, China

**Keywords:** marine metabolites, SZ-685C, nonfunctioning pituitary adenomas, apoptosis

## Abstract

Nonfunctioning pituitary adenoma (NFPA) is one of the most common types of pituitary adenoma. The marine anthraquinone derivative SZ-685C has been isolated from the secondary metabolites of the mangrove endophytic fungus *Halorosellinia* sp. (No. 1403) which is found in the South China Sea. Recent research has shown that SZ-685C possesses anticancer and tumor suppressive effects. The tetrazolium-based colorimetric assay (MTT assay) to investigate the different effect of the marine compound SZ-685C on the proliferation of primary human NFPA cells, rat normal pituitary cells (RPCs) and rat prolactinoma MMQ cell lines. Hoechst 33342 dye/propidium iodide (PI) double staining and fluorescein isothiocyanate-conjugated Annexin V/PI (Annexin V-FITC/PI) apoptosis assays detected an enhanced rate of apoptosis in cells treated with SZ-685C. Enhanced expression levels of caspase 3 and phosphate and tensin homolog (PTEN) were determined by Western blotting. Notably, the protein expression levels of Akt were decreased when the primary human NFPA cells were treated with SZ-685C. Here, we show that SZ-685C induces apoptosis of human NFPA cells through inhibition of the Akt pathway *in vitro*. The understanding of apoptosis has provided the basis for novel targeted therapies that can induce death in cancer cells or sensitize them to established cytotoxic agents and radiation therapy.

## 1. Introduction

Pituitary adenomas (PAs) are one of the most common intracranial tumors with a prevalence of about 80–90/100,000 people [1,2,3]. They can be divided into secretory and non-secretory varieties, with nonfunctioning pituitary adenomas (NFPAs) accounting for about 40% of all pituitary adenomas [4]. They can lead to a range of clinical symptoms usually due to local mass effects such as neurological (headache), ophthalmological (visual field defects and visual loss), and endocrine (hypopituitarism and hyperprolactinemia) symptoms [5,6]. At present, no medical treatment is available to control tumor growth in NFPA [7]. The limited effectiveness of surgery and the risks inherent to radiation therapy warrant the search for new therapeutic options.

In recent years, a wide variety of metabolites has been isolated from marine microorganisms and they have demonstrated huge potential for drug development [8]. They are increasingly attracting the attention of pharmaceutical scientists because of their unique structural properties and specific bioactivity profiles [9,10]. Marine-derived fungi have provided more than 1000 new natural products [11]. *Halorosellinia* sp., a genus of fungi in the family *Xylariaceae*, was first studied for secondary metabolite production by Wyeth [12]. It produce many secondary metabolites, some of them with relevant bioactivity, such as Haloroquinone, a protein kinase B inhibitor [13]. Xia *et al*. [14] isolated the fungus *Halorosellinia* sp., from decayed wood in Hong Kong and a salt lake in the Bahamas, and produced two new anthraquinones.

**Figure 1:**
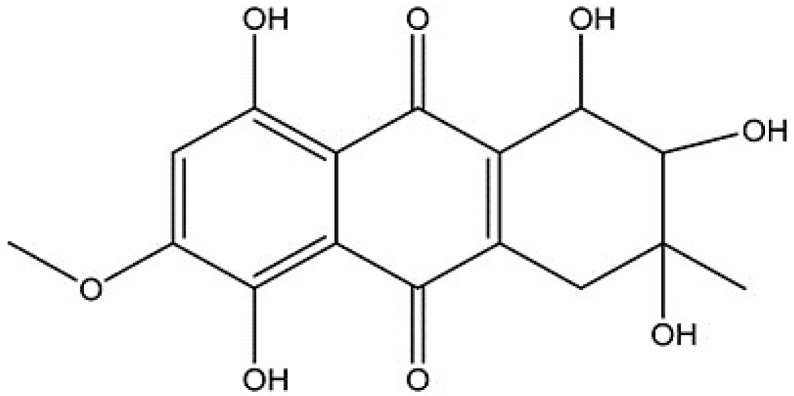
Chemical structure of SZ-685C.

SZ-685C, a novel marine anthraquinone derivative, was isolated from the secondary metabolites of a mangrove endophytic fungus, *Halorosellinia sp.* (No. 1403) from *Kandelia candel* (L.) druce, which is found in the South China Sea (Figure 1). Recent studies have found that this compound can inhibit the growth of a variety of tumor cells, including human glioma, hepatoma, prostate cancer, breast cancer, and nasopharyngeal carcinoma cells [15,16,17].

Apoptosis is the process of programmed cell death, regulated by a complex network of proliferation and survival genes. Akt is a central node in several essential cellular functions and also contributes to tumorigenesis and tumor metastasis [18]. Therefore, drugs that inhibit the Akt pathway may be effective against many human cancers. This study was carried out to evaluate the effectiveness of SZ-685C in the treatment of NFPA. Our results showed that SZ-685C suppressed the Akt pathway and induced apoptosis in primary NFPA cells.

Our current study investigated whether SZ-685C can override chemoresistance by inhibiting Akt signaling in primary human nonfunctioning pituitary adenomas cells.

## 2. Results and Discussion

### 2.1. Identification of Nonfunctioning Pituitary Adenoma (NFPA) Cells Using Electron Microscopy and Immunostaining

**Figure 2:**
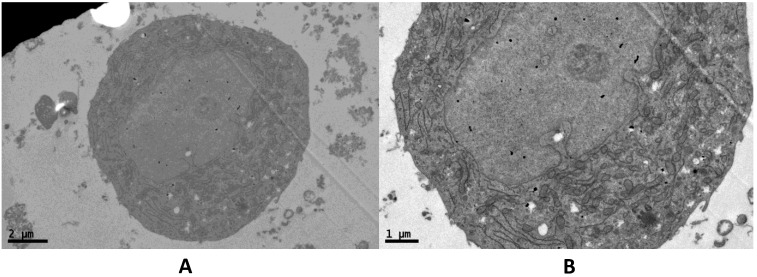
Ultrastructure of an nonfunctioning pituitary adenoma (NFPA) cell. At the ultrastructural level, the large cells possess numerous mitochondria and a few secretory granules, but an otherwise simple cytoplasm. (**A**) Magnification: ×5800, scale bar: 2 μm. (**B**) Magnification: ×9700, scale bar: 1 μm.

We evaluated the ultrastructural and morphometric characteristics of NFPAs by using transmission electron microscopy. We found that an intact cell membrane, large nucleus, abundant euchromatin, and the nucleolus are obvious. In the cytoplasm, the abundance of mitochondria and the small number of secretory granules, allowed us to identify the cultured cells as NFPAs (Figure 2).

**Figure 3:**
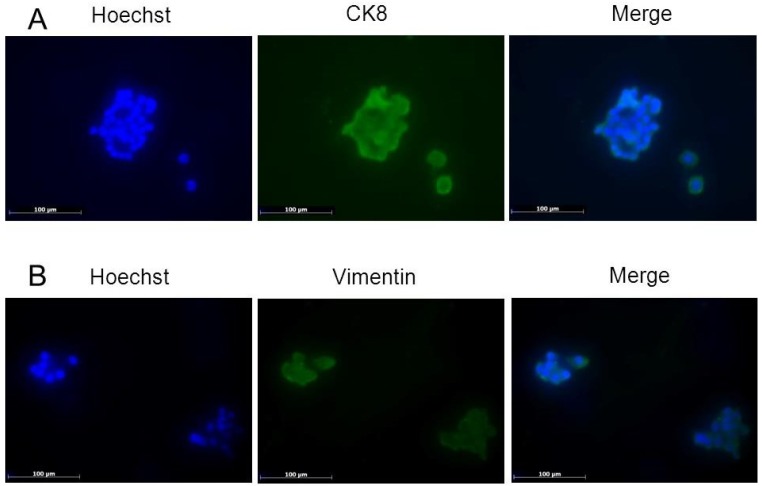
(**A**) CK8 and Hoechst immunofluorescent staining; (**B**) Vimentin and Hoechst immunofluorescent staining. Magnification: × 400, scale bar: 100 μm.

Cytokeratins (CKs) are typically expressed in epithelial cells, whereas vimentin can be found in cells of mesenchymal origin. Co-expression of vimentin and CKs is believed to be the hallmark of epithelial-to-mesenchymal or mesenchymal-to-epithelial transformations of developing tissues. In this study, we analyzed the expression and co-expression of simple epithelial CK8 and vimentin in NFPA cells. The cells’ nuclei were stained with Hoechst 33342 dye, which emits a blue fluorescence, and CK8 and vimentin, which emit a green fluorescence (Figure 3).

### 2.2. Growth Inhibition of Primary Human Nonfunctioning Pituitary Adenoma Cells Induced by SZ-685C

**Figure 4:**
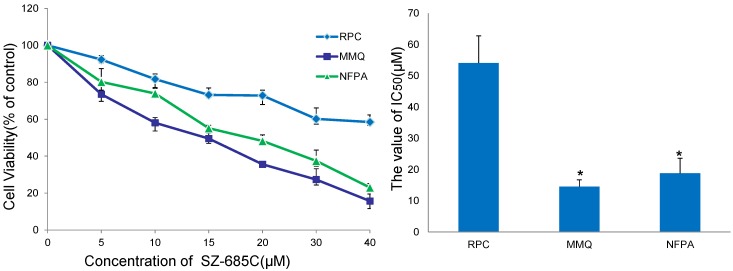
Growth-inhibitory effect of SZ-685C in rat normal pituitary cells (RPCs) and rat prolactinoma MMQ cell lines and primary NFPA cells. All of the cells were treated with the indicated dose of SZ-685C for 24 h and cell viability was determined using an MTT assay.

We used a 3-(4,5-dimethylthiazol-2-yl)-2,5-diphenyltetrazolium bromide (MTT) assay to investigate the effect of the marine compound SZ-685C on the proliferation of human NFPA. Upon treatment of the cells with different concentrations of SZ-685C for 24 h, an obvious inhibitory effect of SZ-685C on cellular proliferation was observed in both the rat normal pituitary cells (RPCs) and rat pituitary adenoma MMQ cell lines. Primary cultures of human nonfunctioning pituitary adenomas cells also exhibited significantly inhibited growth in a dose-dependent manner. We calculated the half maximal inhibitory concentrations (IC_50_) using SPSS 13.0, which revealed that the IC_50s_ of SZ-685C in NFPA, MMQ and RPC cells were 18.76 ± 7.43 μM, 14.51 ± 2.11 μM, and 56.09 ± 5.18 μM, respectively. The difference between the IC_50s_ of the two groups was statistically significant (*p* < 0.05) (Figure 4).

### 2.3. SZ-685C Induces Apoptosis in MMQ Pituitary Adenoma Cells

**Figure 5:**
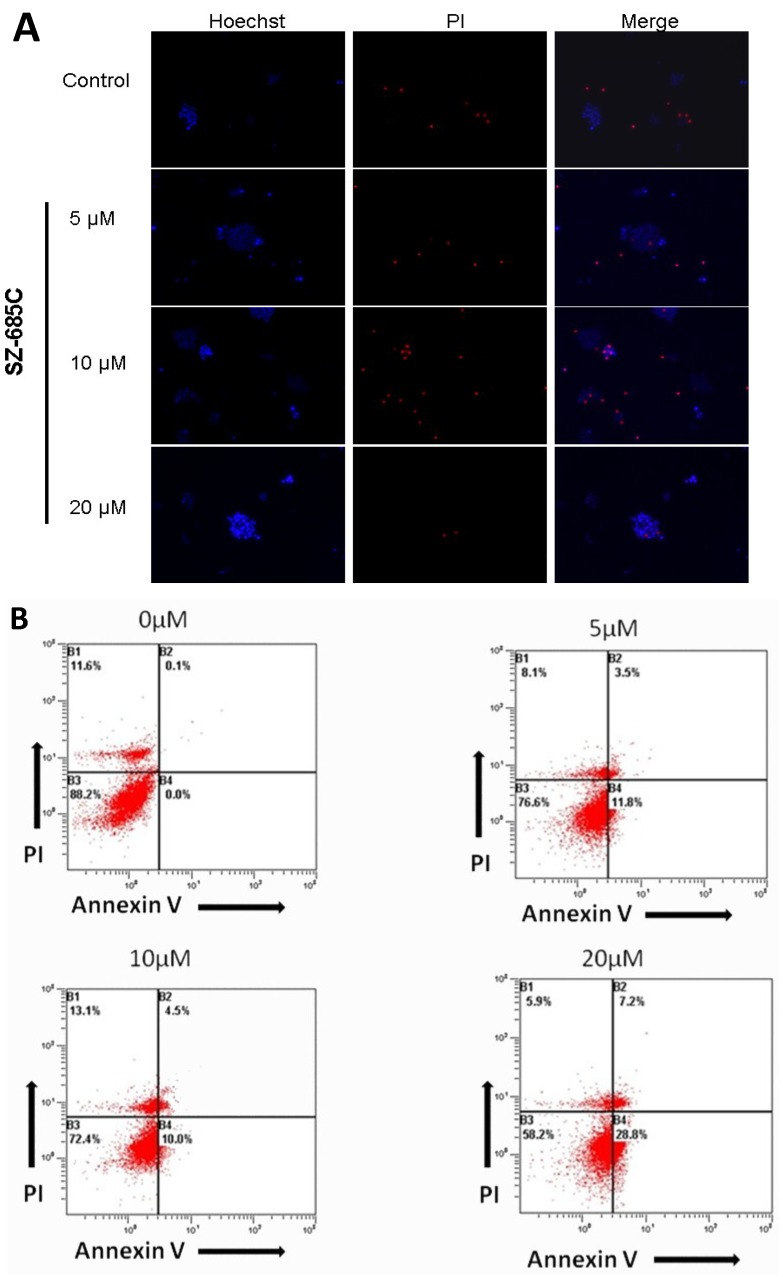

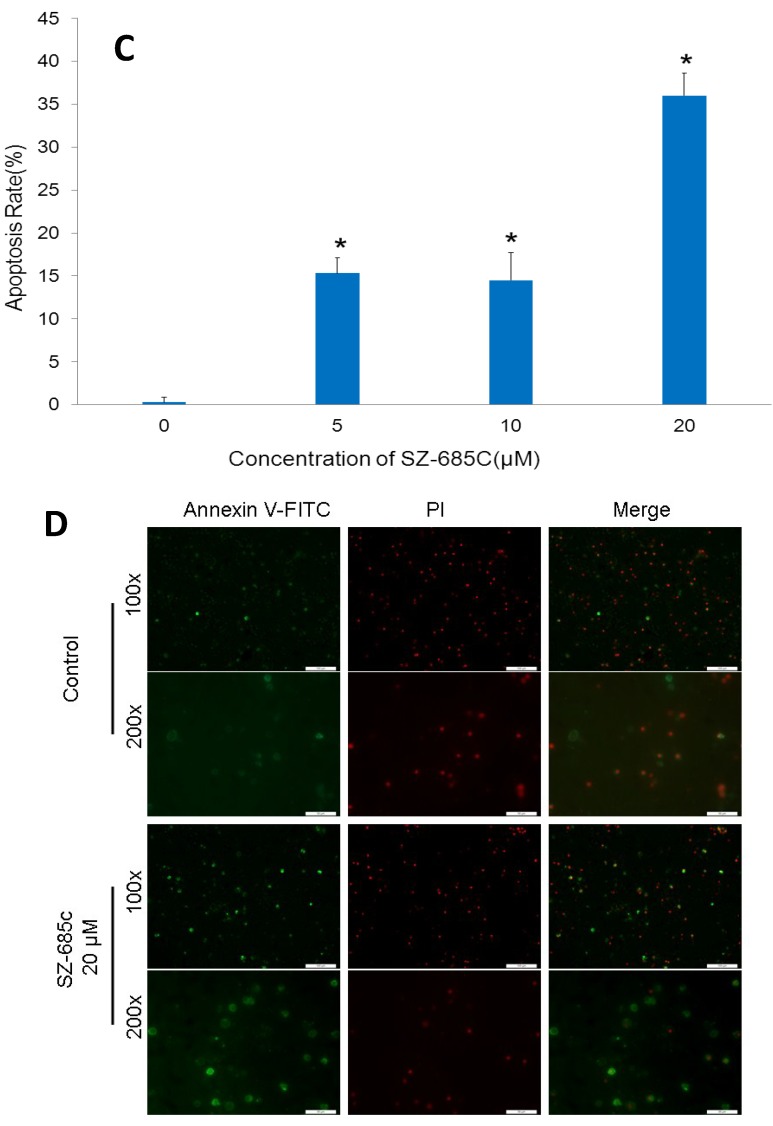
Induction of apoptosis in NFPA cells by SZ-685C. (**A**) Hoechst/propidium iodide (PI) staining and fluorescein isothiocyanate labeled Annexin V (Annexin V-FITC)/PI staining (**D**) of NFPA cells treated with SZ-685C (×100, scale bar: 100 µm; × 200, scale bar: 50 µm); (**B**) Cells were exposed to different concentrations (0, 5, 10, 20 μM) of SZ-685C for 24 h. Cells were then collected and subjected to Annexin V-FITC/PI staining, Hoechst /PI staining and analyzed by flow cytometry (FCM); (**C**) According to the results of FCM (**B**), NFPA cells were treated with SZ-685C for indicated concentration, the apoptosis rate was increased; * *p* < 0.05.

We next investigated whether the inhibition of cell growth induced by SZ-685C in NFPA cells was dependent on the apoptotic pathway. To determine this, we stained MMQ cells with a Hoechst 33342 dye/propidium iodide (PI) double stain after treatment with SZ-685C. The chromatin of cells undergoing apoptosis appears brighter than that of surviving cells due to the red-fluorescence imparted to it by the PI (Figure 5A). A fluorescein isothiocyanate-labeled Annexin V (Annexin V-FITC) and PI apoptosis assay was used to observe programmed cell death by nuclear staining (Figure 5C,D) and flow cytometry (Figure 5B). At the beginning of cellular apoptosis, the membrane phosphatidylserine (PS) flips from the inner face of the plasma membrane to the outer layer, enabling it to bind to Annexin V-FITC. Whereas intact cell membranes exclude PI, dead cells, or those in later stages of apoptosis, are permeable to PI. Flow cytometry showed an increasing apoptosis rate with increasing concentrations of SZ-685C; the 20 µM group exhibited the highest rate of apoptosis at 58.2%.

**Figure 6:**
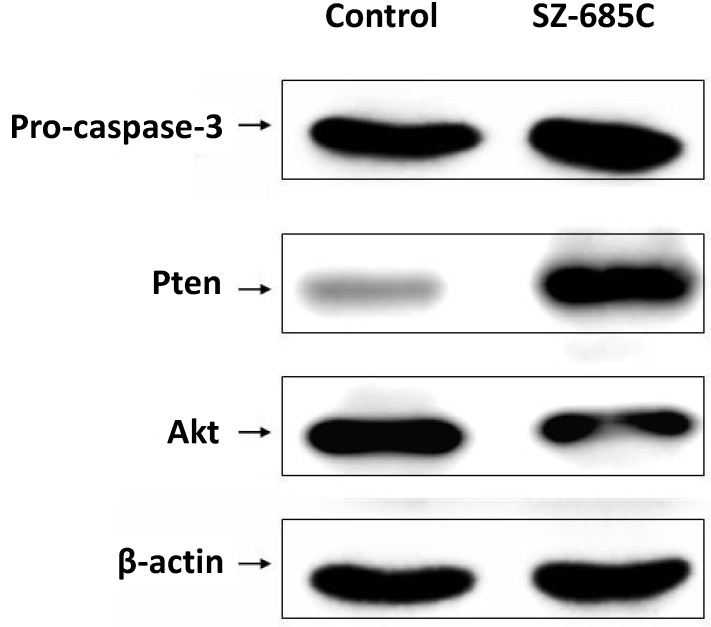
Effects of SZ-685C on apoptosis-related proteins. Western blotting analysis of apoptosis-related proteins incascluding AKT, phosphate and tensin homolog (PTEN), and caspase-3 of the NFPA cells treated with or without 20 µM of SZ-685C. Band intensity was normalized to β-actin expression by densitometry.

Results showed that treatment of NFPA cells with SZ-685C dramatically increased the population of cells stained with both Annexin V and PI (*p* = 0.018, *p* = 0.021), with about 40% of the cells entering apoptosis (Figure 5C). The rates of apoptosis were higher in cells that were treated with SZ-685C than in those which were not. In addition, the expression of both caspase-3 and phosphate and tensin homolog (PTEN) was up-regulated upon treatment with SZ-685C as demonstrated by Western blotting analysis (Figure 6, *p* = 0.004). Notably, the protein expression levels of Akt were decreased when the NFPA cells were treated with SZ-685C (Figure 6).

## 3. Discussion

There is currently no medical treatment for NFPA, and surgery and radiotherapy constitute the cornerstone of therapy. The risks associated with surgical complications and the side effects of radiotherapy underscore the need for new treatment modalities. At present, no NFPA cell lines exist that can be cultured *in vitro*; in this study, we cultured primary human nonfunctioning pituitary adenoma cells from pathological specimens obtained during surgery.

Anthracyclines, so far the most effective anti-cancer agents and commonly used in the clinic, such as Doxorubicin (trade name Adriamycin), Daunorubicin (daunomycin or rubidomycin), Zorubicin (Rubidazone), and Carubicin (carminomycin) [19]. They can against various types of cancer, including breast cancer, leukemias, lymphomas and endometrial carcinoma, *etc*. [20]. However, their severe adverse side effects hampered the successful use of these drugs. Cardiac toxicity is the most serious consequence of anthracycline therapy.

In our previous study, we found that the anthraquinone derivative SZ-685C significantly suppressed the proliferation of the MMQ rat pituitary adenoma cell line and induced apoptosis through the downregulation of miR-200c [21]. Caspases play an important role in cell apoptosis. Activation of caspases appears to be directly responsible for many molecular and structural changes involved in apoptosis. The caspase-dependent process of programmed cell death can proceed through two pathways of anticancer drug-induced apoptosis: the extrinsic (death receptor) pathway and the intrinsic (mitochondrial) pathway. Caspase-3 is one of the downstream effectors of the caspase family, and is thought to be involved in both the mitochondrial apoptotic pathway and the death receptor pathway. In this study, we observed that the SZ-685C induced apoptosis in NFPA cells is accompanied with increased caspase activity. Taken together, our data strongly suggest that SZ-685C-induced cytotoxicity may be mediated through caspase-dependent apoptosis.

The Akt signaling pathway plays a crucial protective role against programmed cell death in cancer cells. Akt is an important oncogenic protein that can regulate various important processes in the cell by influencing the participation of other proteins. We investigated whether inhibition of the Akt signaling pathway had an effect on its downstream targets in SZ-685C-treated NFPA cells. Zhu *et al.* reported that SZ-685C induces apoptosis in adriamycin-resistant human breast cancer cells both *in vitro* and *in vivo*, and that it exerts these antitumor effects through suppressing Akt signaling [17]. Xie *et al.* found that SZ-685C inhibits the growth of six tumor cell lines, including human glioma, hepatoma, prostate cancer, and breast cancer (IC_50_ = 3.0–9.6 μM). They also found that SZ-685C inhibits tumor growth in nude mice by inducing apoptosis via the Akt/FOXO pathway [15]. Wang *et al.* reported that SZ-685C displayed a potent cytocidal effect in both radiosensitive and radioresistant human nasopharyngeal carcinoma (NPC) cells via the miR-205-PTEN-Akt pathway [16].

We have demonstrated that SZ-685C could destroy tumor cells via activating caspase-3-mediated apoptotic mechanisms through suppressing the phosphorylation of Akt. Our study shows that the expression of Akt-related genes is decreased after treatment with SZ-685C.

Our experiments were limited by the scarcity of NFPA samples and their inherently slow growth rate. We need to continue investigating the mechanism of SZ-685C-induced apoptosis and collecting more biological tissue samples from the clinic. Understanding the mechanism of apoptosis could provide the basis for novel targeted therapies that can induce death in cancer cells or sensitize them to established cytotoxic agents and radiation therapy [22].

## 4. Experimental Section

### 4.1. Preparation of SZ-685C

SZ-685C was prepared and purified from mangrove endophytic fungus (No. 1403), as previously reported [15]. The compound was dissolved in dimethyl sulfoxide (DMSO, 2.5‰ of final concentration) to a concentration of 1 mM as a stock solution and diluted as indicated, according to experimental requirements.

### 4.2. Cell Culture

RPC cells (ScienCell, Logan, UT, USA, NO: R1200), an adherent cell line, were cultured in RPMI 1640 medium (Hyclone, Logan, UT, USA) supplemented with 10% fetal bovine serum (Gibco, New York, NY, USA), 100 mg·mL^−1^ streptomycin and 100 units·mL^−1^ penicillin (Invitrogen, Grand Island, NY, USA). The rat pituitary adenoma cell lines, MMQ and GH3, were purchased from Xie-he Bank (Beijing, China). They were cultured in DMEM/F-12 medium (Hyclone, USA) supplemented with 15% horse serum (Gibco, USA) and 2.5% FBS (Gibco, USA). All of the cell lines were cultured at 37 °C in a humidified atmosphere of 5% CO_2_ [23].

### 4.3. Pituitary Adenoma Samples

All of the pituitary adenoma samples were obtained from the Department of Neurosurgery and Pituitary Tumor Center of the First Affiliated Hospital, Sun Yat-sen University (Guangzhou, China). The local ethical committee approved the patients’ treatment, and all of the participants provided informed written consent.

### 4.4. Cell Viability Assay

MTT assays were used to assess cell viability. NFPA cells and MMQ cells were seeded in 96-well plates (Corning, New York, NY, USA) at a density of 5 × 10^4^ cells per well and cultured in serum-free DMEM/F-12 medium for 24 h. Then, each well was stimulated with the indicated concentration of SZ-685C (0–40 µM) for 24 h. The adherent cell line, RPC, was seeded and stimulated by the drug as described in the NFPA cell protocol. Subsequently, 10 μL MTT (dissolved in phosphate-buffered saline (PBS) at a pH of 7.4 (5 mg·mL^−1^; Sigma, St. Louis, MO, USA) was added to each well and incubated for 4 h at 37 °C in a humidified atmosphere of 5% CO_2_. Next, 100 µL acidified isopropyl alcohol was added. Then, the optical density (OD) was measured by a Sunrise microplate reader (TECAN, Männedorf, Switzerland) at 570 nm with a reference wavelength of 630 nm. The IC_50_ was analyzed by SPSS 13.0 (SPSS, Chicago, IL, USA). The cell viability rate was calculated as follows: Cell viability rate = ((OD of treated cells − OD of blank)/(OD of control cells − OD of blank)) × 100%.

### 4.5. Hoechst 33342/PI Dye Assay

Apoptotic cells were detected by a Hoechst 33342/PI dye assay. The cultured primary NFPA cells were harvested and seeded in 6-well plates (4 × 10^5^/well) and incubated with the indicated concentrations of SZ-685C for 24 h. Then, the cells were collected and washed three times with PBS, and stained using 10 μL Hoechst 33342 (5 μg/mL; Keygen Biotech, Nanjing, China) and 5 μL PI (2.5 μg/mL; Keygen Biotech, Nanjing, China) in PBS. Finally, the apoptotic cells were observed under the fluorescence microscope (Axio Observer Z1, Peabody, MA, USA).

### 4.6. Annexin V-FITC/PI Staining

The apoptosis rate of MMQ cells was determined using Annexin V-FITC and PI staining (eBioscience, San Diego, CA, USA). NFPA cells were treated with SZ-685C at the indicated concentration for 24 h in 6-well plates (2 × 10^5^ cells/mL). Then, cells were collected and washed with PBS three times and resuspended in 500 μL binding buffer. Next, 5 μL Annexin V-FITC and 10 μL PI were added to each sample, and the samples were incubated in the dark for 10 min. Cell fluorescence was analyzed immediately after staining with the fluorescence microscope and flow cytometer (Epics XL-MCL, Beckman Coulter, Indianapolis, IN, USA). The FITC signal detector (FL1) and PI staining signal detector (FL3) were used to detect the cells with the flow cytometer (Ex = 488 nm; Em = 530 nm).

### 4.7. Western Blotting

Total protein was extracted from NFPA cells after treatment at different concentrations of SZ-685C for 24 h using a lysis buffer (Applygen, Beijing, China). The protein concentrations were measured using a Bio-Rad protein assay kit (Bio-Rad Laboratories Inc., Hercules, CA, USA), and 30 µg of total protein was subjected to SDS-PAGE immunoblotting analysis. Polyvinylidene fluoride (PVDF) membranes (Bio-Rad Laboratories Inc., USA) were blocked with 5% non-fat milk in TBST (Tris-buffered saline containing 0.1% Tween 20; Sigma, USA) for 1 h at room temperature. After washing twice with TBST (5 min each), the membrane was incubated with caspase-3 (1:1000; ABclonal, Cambridge, MA, USA), Akt (1:1000; ABclonal, USA), PTEN (1:1000; Cell Signaling Technology, Danvers, MA, USA), and β-actin primary antibodies (1:2000; Santa Cruz, Dallas, TX, USA) at 4 °C overnight. The next day, the membrane was incubated with a horseradish peroxidase-conjugated secondary antibody (ProteinTech, Chicago, IL, USA) for 1 h at room temperature, and signal detection was performed using the ultrasensitive ECL Plus Detection Reagent (Applygen Technologies Inc, Beijing, China).

## 5. Conclusions

Our data suggest that SZ-685C may be a potentially promising Akt inhibitor and anti-cancer agent for the treatment of NFPA (Nonfunctioning pituitary adenoma).
